# Efficient Fabrication of Human Corneal Stromal Cell Spheroids and Promoting Cell Stemness Based on 3D-Printed Derived PDMS Microwell Platform

**DOI:** 10.3390/biom15030438

**Published:** 2025-03-19

**Authors:** Yuexi Chen, Jianing Gu, Zekai Cui, Xihao Sun, Yuqin Liang, Chunwen Duan, Xiaoxue Li, Zhanyu Su, Bo Zhang, Jiansu Chen, Zheng Wang

**Affiliations:** 1 The First Clinical Medical College, Jinan University, Guangzhou 510632, China; 2 Guangzhou Aier Eye Institute, Guangzhou 510071, China; 3 Aier Academy of Ophthalmology, Central South University, Changsha 410015, China; 4 Key Laboratory of Regenerative Medicine of Ministry of Education, Jinan University, Guangzhou 510632, China

**Keywords:** human corneal stromal cells, SMILE, three-dimensional spheroid culture, PDMS, stem cells, stemness, pluripotency

## Abstract

Spherical culture could promote the plasticity and stemness of human corneal stromal cells (hCSCs). Here, we introduce a novel three-dimensional (3D) cell culture system based on a polydimethylsiloxane (PDMS) microwell platform composed of many V-bottom microcavities to generate human corneal stromal cell spheroids and promote cell stemness. We isolated hCSCs from SMILE-derived lenticules and maintained their physiological phenotype by culturing them in a medium supplemented with human corneal stromal extract (hCSE). Utilizing a PDMS microwell platform fabricated through 3D printing technology, we successfully generated 3D corneal stromal cell spheroids (3D-CSC) with uniform size and stable structure, exhibiting increased expression of pluripotency factors, including OCT4, NANOG, SOX2, KLF4, and PAX6. Furthermore, the iPS supernatant of E8-conditioned medium (E8-CM) significantly enhanced the stemness properties of these cells. RNA sequencing and proteomics analyses revealed that 3D-CSCs exhibited superior proliferation, differentiation, cell adhesion, migration, and neurogenesis compared to traditional monolayer cultures, underscoring the role of biophysical cues in promoting hCSCs stemness. In summary, this study presents an effective 3D cell culture platform that mimics the in vivo microenvironment, facilitating the enhancement of stemness properties and providing valuable insights into corneal tissue engineering and regenerative medicine, particularly for treating corneal opacities and diseases.

## 1. Introduction

The corneal stromal cells (CSCs, also called Keratocytes), physiological cells of the corneal stroma, play an indispensable role in maintaining corneal transparency, structure, and physiological function. In their quiescent state, these cells exhibit a flattened, dendritic morphology and communicate via gap junctions. Keratocytes actively synthesize and maintain the extracellular matrix (ECM), which consists of various collagens, proteoglycans, and glycoproteins [1,2]. The precise synthesis and orderly assembly of collagen fibers within the ECM are essential for the biomechanical properties and optical clarity of the corneal stroma [3,4]. Upon corneal injury, corneal stromal cells (CSCs) undergo substantial phenotypic and functional changes, including apoptosis, proliferation, and ECM remodeling [5]. Quiescent keratocytes can differentiate into fibroblasts and myofibroblasts, characterized by their contractile properties and excessive ECM synthesis [6,7,8]. This transdifferentiation can result in disorganized ECM deposition and fibrotic scarring, leading to corneal opacity and vision impairment—common complications following corneal injuries or surgeries.

Cell therapy, which involves transplanting healthy cells to damaged sites to replace dysfunctional cells, has been proven to be an effective approach for treating corneal diseases. However, identifying cell sources with self-renewal capabilities remains one of the major challenges in cell therapy. Cells derived from the cornea stroma have demonstrated pluripotency potential [9,10]. Additionally, promoting the pluripotency and stemness of adult CSCs into stem-like cells, especially corneal stromal stem cells (CSSCs), presents significant implications for regenerative medicine. Firstly, CSSCs exhibit multilineage differentiation potential, capable of differentiating into corneal cells, osteocytes, adipocytes, and neural cells, with regenerative properties akin to embryonic stem cells [11]. Moreover, CSSCs can remodel pathological stromal tissue, suppress inflammation, and restore transparency, making them a promising therapeutic option for corneal scarring and related diseases [12,13,14,15]. Since CSSCs can be obtained from patient-derived biopsies, they offer the potential for autologous stem cell therapy, circumventing immune rejection and mitigating ethical concerns associated with allogeneic transplantation.

The 3D spheroid culture has become increasingly popular in recent years as it is more relevant to the in vivo condition. Many studies have shown that compared to traditional monolayer (2D) cultures, three-dimensional (3D) cultures better mimic the native microenvironment by preserving ECM-cell interactions, promoting cell-to-cell communication [16,17,18], enhancing cell survival and post-transplantation viability [17,18], maintaining stemness and differentiation potential [17,19], and improving drug response [17]. Research showed that 3D culture is crucial for the chondrogenesis of the mesenchymal stem (MSC) cells and also enhances their osteogenic differentiation, as well as their multilineage potential and survival capacity [18,20,21], and the upregulation of pluripotency-associated factors including Oct4, Sox2, and Nanog [20,22]. Chang et al. presented that a chitosan 3D cell culture system can sustain the pluripotency of human induced pluripotent stem cells (hiPSCs) and facilitate their differentiation over long-term culture periods, lasting up to 365 days [23]. However, the efficient fabrication of human corneal stromal spheroids and the promotion of cell stemness remains a challenge. The process seems to have low efficiency and is likely influenced by various factors, including age, type, and source of the cells that are utilized [24].

Polydimethylsiloxane (PDMS) is a versatile substrate with tunable stiffness and roughness, but its low surface energy and hydrophobicity can impede cell attachment while promoting cell aggregation into spheroids. Physical modulation of the PDMS surface, such as altering stiffness or creating micro/nano topography, enhances cell responses by influencing cell-substrate interactions [25,26,27]. These biophysical cues can significantly affect cell behavior, morphology, and cytoskeleton arrangement, thereby promoting cell growth and differentiation [28,29,30]. Our previous work revealed that PDMS could promote keratocyte reprogramming, possibly due to changes in the regulation of actin cytoskeleton dynamics and the activation of TGFβ/BMP and HIF-1 signaling pathways that may induce alterations in mechanotransduction, mesenchymal-to-epithelial transition (MET), and hypoxia adaptation [31]. Furthermore, we demonstrated that a PDMS microwell platform improves the robustness and standardization of in vitro retinal organogenesis and retinal organoid cultures [32].

To further enhance the stemness properties of hCSCs, we explored the use of an induced pluripotent stem (iPS) cell-conditioned medium (iPS-CM) as a culture supplement. Studies have shown that iPS-CM contains bioactive factors that influence cell function [33,34]. Recent reports highlight its protective effects in acute lung injury (ALI) [35], cardiac repair post-infarction [36], and alleviating inflammation in bacterial-induced lung injury [37]. Our previous research also found that iPS-CM enhances the characteristics of retinal pigment epithelium (RPE) sheets by promoting cell density and function, which are critical for RPE therapy [38].

Small incision lenticule extraction (SMILE) is a widely used refractive surgery that generates corneal stromal lenticules, which are often discarded as medical waste. However, these lenticules serve as an ideal source of human corneal stromal cells (hCSCs), as they are free from contamination by corneal epithelial and endothelial cells. Furthermore, our previous work demonstrated that culturing CSCs with porcine corneal stromal extract (pCSE) in a low-concentration fetal bovine serum (FBS) environment effectively preserves their physiological phenotype, prevents fibrosis, and promotes proliferation [39]. These advantages provide a robust approach to obtaining high-quality seed cells for corneal tissue engineering and cell-based therapies.

In this study, we efficiently utilized SMILE-derived corneal stromal lenticules as a resource for isolating and expanding hCSCs in vitro. By leveraging a novel spheroid culture system of a 3D-printed PDMS microwell platform, we successfully generated homogeneous and adherent 3D-hCSCs with enhanced stemness characteristics. Our research has established a promising foundation for elucidating the potential applications of CSCs spheroids in tissue engineering and cell therapy in corneal regenerative medicine.

## 2. Materials and Methods

### 2.1. Ethics Statement

The human corneal stromal lenticules used in this study were obtained from SMILE surgery and approved by the Ethics Committee of Aier Eye Hospital Group (Changsha, Hunan, China, 2023KYPJ009). All procedures adhered to the principles outlined in the Declaration of Helsinki. Written informed consent was obtained from all patients, who were healthy individuals with no history of systemic or ocular diseases.

### 2.2. Isolation and Culture of Human Corneal Stromal Cells

Human corneal stromal extract (hCSE) was prepared following the method described by Li et al. [39]. Human corneal stromal lenticules were collected from SMILE surgery, immersed in a DMEM/F12 and glycerol mixture (1:1 vol/vol), and stored at −80 °C for one week for deactivation. The lenticules were then homogenized into a tissue slurry using liquid nitrogen. The upper protein extract was collected, filtered, sterilized, and stored for further use.

Human corneal stromal cells (hCSCs) were harvested from fresh SMILE-derived lenticules immediately after surgery (Changsha Aier Eye Hospital, Changsha, China). After rinsing in sterile PBS, the lenticules were digested with 1 mg/mL type I collagenase (Sigma-Aldrich, St. Louis, MO, USA) at 37 °C overnight. The cells were harvested by centrifugation and then seeded onto 6-well plates coated with collagen I (Corning Incorporated, Corning, NY, USA). Subsequently, they were cultured in a low-serum RIFA + hCSE medium, which contained the following components: 10 μM ROCK inhibitor Y-27632 (TargetMol), 10 ng/mL Insulin-Transferrin-Selenium (ITS, Sigma-Aldrich, St. Louis, MO, USA), 10 ng/mL fibroblast growth factor-2 (FGF2, PeproTech, Cranbury, NJ, USA), 1 mmol/L ascorbate 2-phosphate (Sigma), and 5 μg/mL soluble hCSE. The RIFA + hCSE medium was based on DMEM/F12 (Gibco, Waltham, MA, USA) and supplemented with 0.5% fetal bovine serum (FBS, Sigma), 1% penicillin-streptomycin (Gibco), and the aforementioned additives. The control normal medium (NM) contained DMEM/F12, 10% FBS, and 1% penicillin-streptomycin. Cultures were maintained at 37 °C with 5% CO_2_, with media changes every 2–3 days. Cells were passaged upon reaching 80–90% confluence, and only passage 2 (P2) hCSCs were used for further experiments.

### 2.3. Culture of Urine-Induced Pluripotent Stem Cells (iPS) and Preparation of iPS-Conditioned Medium

The iPS cells were cultivated following established protocols [40]. Six-well plates were pre-coated with a 1% (*v*/*v*) Matrigel (Corning, Corning, NY, USA) solution for 30 min before seeding the iPS cells in Essential 8 (E8) medium (Gibco, Waltham, MA, USA). When cells reached 50–80% confluence, the supernatant was collected, filtered through a 0.22-micron filter, and stored at −80 °C. The iPS-conditioned medium (iPS-CM) was prepared by mixing the filtered supernatant with E8 medium at a 1:1 ratio.

### 2.4. Preparation of the 3D-Printed PDMS Microwell Platform

**Figure 1:**
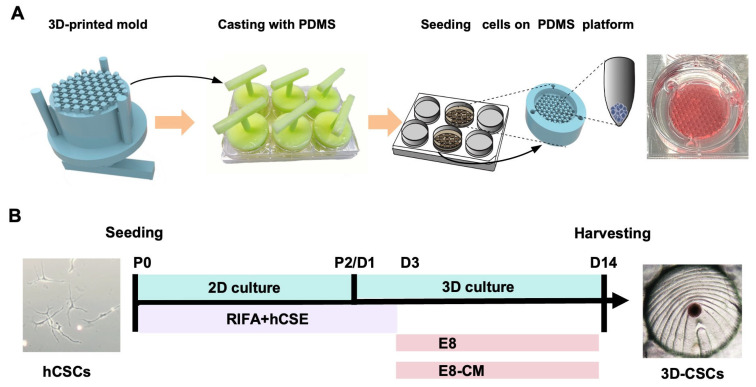
Preparation of the PDMS Microwell Platform and generation and culture of 3D-CSCs on PDMS. (**A**) Flowchart of fabricating a PDMS V-shaped microwell using the 3D-printed mold. (**B**) Timeline and protocol for the culture from hCSCs to 3D-CSC.

The PDMS microwell platform was fabricated based on the method reported by Sun et al. [32]. A master positive mold and Sylgard 184 silicone elastomer (Dow Corning, Midland, MI, USA) were employed to create the platform. The master positive mold was constructed via 3D printing using a photosensitive resin. Subsequently, the culture mold was fabricated by casting a complementary PDMS mold. For PDMS preparation, a 10:1 ratio of base component to curing agent was mixed at 500 RPM for 20 min. Air bubbles were removed using a magnetic stirrer. The 3D-printed mold was placed in a 6-well plate, filled with 5 mL of PDMS prepolymer, and thermally cured at 70 °C for 3–5 h. Once hardened, the PDMS was carefully removed and sterilized at 121 °C for 30 min before being arranged in 6-well plates for use. The resulting PDMS microwell platform, designed for cell culture, features multiple V-bottom cylindrical microwells (Figure 1A).

### 2.5. Generation and Culture of 3D-CSCs

As described earlier, hCSCs were cultured on tissue culture plates (TCP) using RIFA + hCSE medium up to P2. Subsequently, a 3D spherical culture was performed to generate 3D-CSCs using the method described by Sun et al. [32]. To be specific, the PDMS microwell platform was placed in a single well of a 6-well plate, and hCSCs at P2 were digested with Accutase (Merck Millipore, Boston, MA, USA) and seeded onto the PDMS microwell platform at a density of approximately 1.5 × 10^6^ cells per well. Plates were centrifuged at 100 RPM for 10 min to allow cell sedimentation. For the first 2 days of 3D spheroid culture, the medium was maintained as RIFA + hCSE. On day 3, the medium was changed to E8-CM/E8 medium and maintained for the next 12 days to enhance stemness, resulting in a total culture period of 14 days. All media was refreshed every two days. To optimize spheroid formation and promote the stemness of 3D-CSCs, we compared three different culture conditions during the 3D culture: E8-CM medium (E8 medium supplemented with iPS-conditioned medium) on PDMS (E8-CM group) and E8 medium on PDMS (E8 group). Control groups were CSCs cultured on TCP before spheroid formation (TCP group). To further enhance the experimental rigor, we introduced an additional control group of CSCs cultured in E8-CM on TCP for two weeks, designated as the TCP-CM group. (Figure 1A,B)

### 2.6. Live/Dead Staining

On day 14, 3D-CSCs viability was assessed using the LIVE/DEAD™ cell imaging kit (Thermo Fisher Scientific, Waltham, MA, USA). Samples were incubated at room temperature for 15 min and imaged using a fluorescence microscope (IX71, Olympus, Tokyo, Japan) at excitation wavelengths of 488 nm and 570 nm. Three biological replicates were performed.

### 2.7. Quantitative Real-Time Polymerase Chain Reaction (qPCR)

Total RNA was extracted using the RNA Extraction Kit (Accurate Biology, Changsha, China), and cDNA synthesis was conducted using the ReverTra Ace qPCR RT kit (TOYOBO, Osaka, Japan). Gene-specific primers were designed via Primer-BLAST (NCBI) and are listed in Appendix A Table A1. qPCR was performed on a CFX 96 system (Bio-Rad, Hercules, CA, USA), with gene expression normalized to GAPDH levels. Each sample had three biological replicates, and transcript changes were analyzed using the 2^−∆∆Ct^ method.

### 2.8. Immunofluorescence Staining

Cells were fixed, permeabilized, blocked, and incubated overnight at 4 °C with primary antibodies against ALDH3A1, Lumican, Fibronectin, α-SMA, Nanog, Sox2, Klf4, Pax6, Nestin, NeuN, SPHK1, SPP1, and TIMP1. The primary antibody information is listed in Appendix A Table A2. Secondary antibodies (Alexa Fluor 488/594/647, Invitrogen, San Diego, CA, USA) were applied for 1 h at room temperature, followed by DAPI nuclear staining. Imaging was performed with an LSM800 confocal microscope (ZEISS, Oberkochen, Germany).

### 2.9. Western Blot Analysis

Proteins were extracted using a Beyotime protein extraction solution and quantified using a BCA Protein Assay Kit (Solarbio, Beijing, China). Proteins were separated via SDS-PAGE, transferred to PVDF membranes, and blocked for 2 h at room temperature. Membranes were incubated overnight at 4 °C with primary antibodies against SPHK1, SPP1, TIMP1, and NeuN. The primary antibody information is listed in Appendix A Table A2. After washing, membranes were incubated with HRP-conjugated secondary antibodies (1:3000, Bios, Beijing, China), followed by visualization using SuperSignal™ West Femto Maximum Sensitivity Substrate (Thermo Fisher Scientific, Waltham, MA, USA).

### 2.10. RNA-Seq Analysis

Total RNA was extracted from 2D and 3D CSCs and analyzed via RNA sequencing (BGI Biotech Co., Ltd., Shenzhen, China). The raw sequencing data were processed using SOAP [41] to remove reads containing sequencing adaptors, low-quality sequences, and unknown bases. The cleaned reads were then mapped to the reference transcriptome using Hisat2 [42], and Bowtie2 [43] was used to align the reads to the reference coding gene set. Gene expression levels were normalized using RPKM values. Differentially expressed genes (DEGs) were identified using |log2 (fold change)| > 2 and FDR < 0.05. The correlation coefficient between gene expression levels was calculated, and a correlation heat map was generated. Pathway enrichment analysis primarily utilized the Kyoto Encyclopedia of Genes and Genomes (KEGG) database, and a paired t-test was employed to compare the two groups.

### 2.11. Proteomics Analysis

This study utilized the direct DIA method to acquire data from six samples, including both Group 2D and Group 3D. Proteins were extracted with 1% SDS and 8 M urea buffer, then digested with trypsin using the iST kit (PreOmics, Beijing, China). Cleaned peptides were reconstituted in 0.1% FA and analyzed on a timsTOF Pro2 mass spectrometer (Bruker, Billerica, MA, USA) coupled with an UltiMate 3000 LC system (Thermo Fisher, Waltham, MA, USA). Peptides were separated on a C18 column. Data were acquired in diaPASEF mode with 22 × 40 Th windows from m/z 349 to 1229. Data were processed using Spectronaut 18 with default settings. Carbamidomethyl (Cys) was set as a fixed modification, and oxidation (Met) as a variable modification. Normalization was performed using Local normalization, and peptides passing the 1% Q-value cutoff were quantified by MaxLFQ. The FDR was controlled at 1% for both precursors and proteins. The functions and classifications of the proteins were analyzed by searching against several databases, including Gene Ontology (GO) and the Kyoto Encyclopedia of Genes and Genomes (KEGG) [44]. Appropriate thresholds (Fold Change > 1.5, FDR < 0.05) were established to screen for differentially expressed proteins (DEPs). These proteins were subjected to functional annotation and pathway enrichment analysis to explore their potential biological significance and related signaling pathways.

### 2.12. Statistical Analysis

Data are expressed as mean ± SD. Group comparisons were performed using Student’s t-test (SPSS 18.0, GraphPad Prism 9.3). Statistical significance was set at *p* < 0.05.

## 3. Results

### 3.1. hCSCs from SMILE-Derived Lenticules

**Figure 2:**
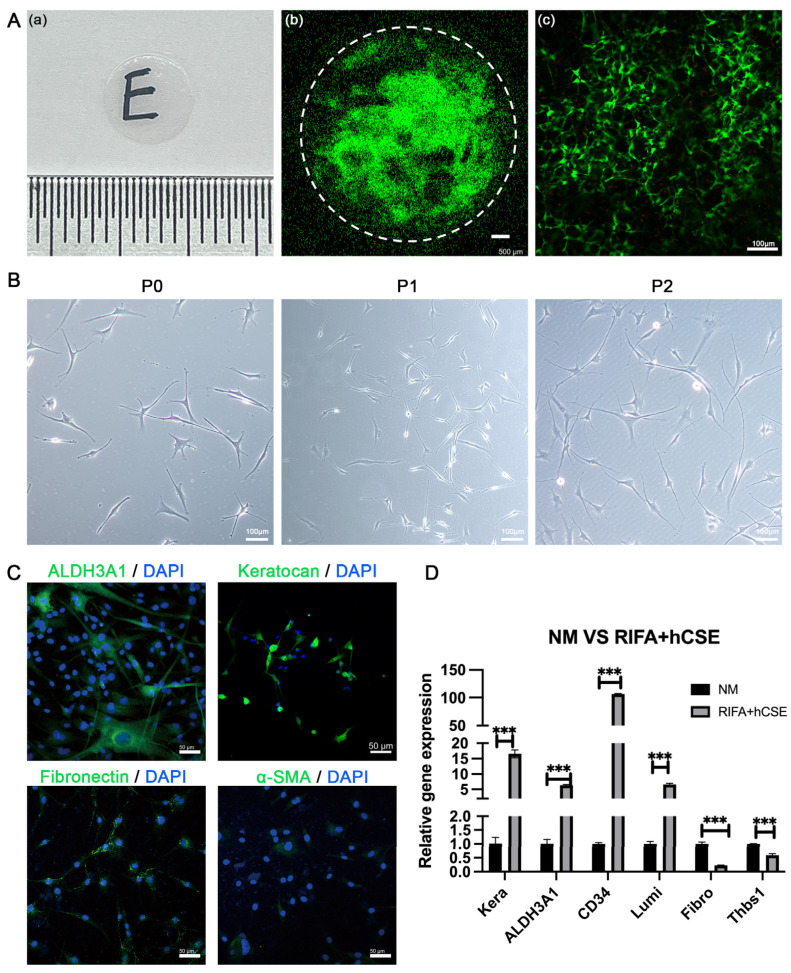
Characteristics of hCSCs isolated from SMILE-derived lenticules. (**A**-**a**) Morphology of fresh lenticules from SMILE post-surgery. (**A**-**b**,**c**) Live/dead cell staining demonstrated that the cells in the fresh lenticules maintained good viability, with a predominant presence of green-stained viable cells and only a few red-stained dead cells scattered sparsely. (**B**) Light microscopy images revealed that hCSCs cultured in RIFA + hCSE medium exhibited dendritic or stellate shapes at P0, P1, and P2. (**C**) Immunofluorescence (IF) analysis showed that hCSCs strongly expressed keratocyte markers (ALDH3A1 and Keratocan, marked green) while weakly staining for myofibroblast markers (Fibronectin and α-SMA, marked green) in RIFA + hCSE medium. Scale bars: 50 μm. (**D**) qPCR analysis confirmed that hCSCs expressed higher levels of keratocyte markers (ALDH3A1, Keratocan, CD34, and Lumican) and lower levels of fibroblast markers (Fibronectin and Thbs1) compared to fibroblasts. (*** *p* < 0.001). *n* = 3 for all.

Corneal stromal lenticules, freshly extracted post-SMILE procedure, were around 6.5 mm in diameter (Figure 2A). Primary hCSCs were successfully isolated and cultured from these lenticules using collagenase I digestion. Live/dead cell staining confirmed the viability of cells within the fresh lenticules, with a predominant presence of green-stained viable cells and only a small fraction of red-stained dead cells, which were sparsely distributed (Figure 2A). Morphologically, the primary hCSCs exhibited a dendritic or stellate shape, extending protrusions in multiple directions to establish connections with neighboring cells (Figure 2B). Monolayer-cultured hCSCs reached confluence within 5–7 days, after which they were passaged. For this study, only passage two (P2) hCSCs were utilized.

Immunofluorescence (IF) staining revealed that hCSCs cultured in RIFA + hCSE medium strongly expressed quiescent corneal keratocyte markers, ALDH3A1, and Keratocan while showing weak staining for corneal fibroblast markers, Fibronectin, and α-SMA (Figure 2C). Furthermore, gene expression analysis demonstrated significantly higher levels of ALDH3A1, CD34, Keratocan, and Lumican compared to fibroblasts cultured in the control normal medium (NM) containing 10% FBS (6.34-fold, 106.47-fold, 16.55-fold, and 6.63-fold, respectively). In contrast, the expression of fibrotic markers Fibronectin and Thbs1 was significantly lower in the RIFA + hCSE group (0.23-fold and 0.59-fold, respectively) (Figure 2D). These findings indicate that hCSCs derived from SMILE lenticules retain the morphological and phenotypic characteristics of quiescent corneal stromal cells when cultured in the RIFA + hCSE medium.

### 3.2. Generation and Enhancement of hCSC Stemness in 3D-CSCs

**Figure 3:**
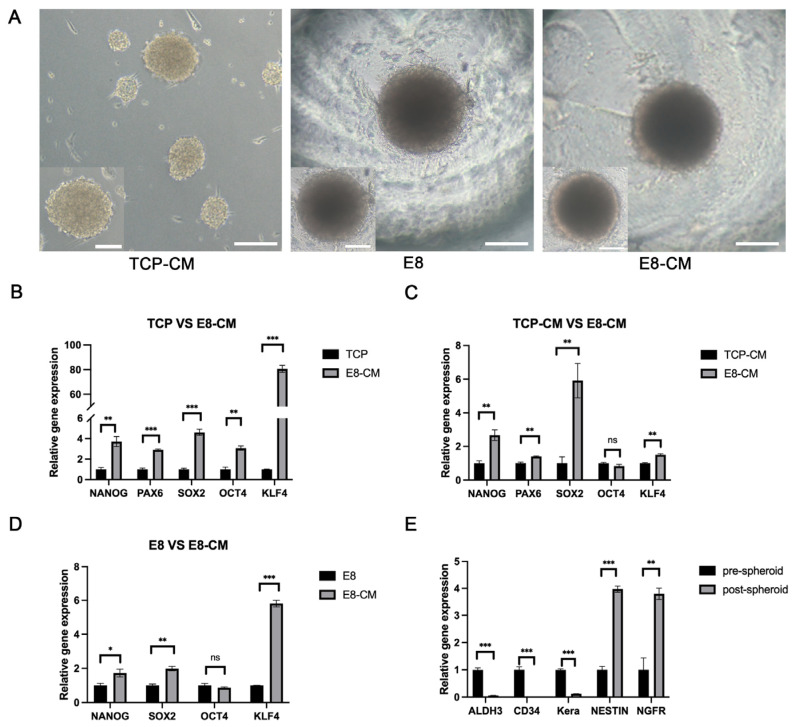
The PDMS microwell platform with E8-CM facilitates the formation of 3D-CSCs. (**A**) Bright-field images of 3D-CSCs under various culture conditions. (**B**–**D**) qPCR analysis demonstrated that the expression of stem cell marker genes (SOX2, PAX6, OCT4, NANOG, KLF4) in 3D-CSCs cultured in E8-CM was significantly higher than in other culture conditions. (**E**) Additionally, keratocyte markers (ALDH3A1, KERA, and CD34) were downregulated, while neural crest stem cell markers were upregulated in 3D-CSCs compared to pre-spheroids. (* *p* < 0.05; ** *p* < 0.01; *** *p* < 0.001, *ns* means not significant, *n* = 3 for (**B**–**E**)). Scale bars: 100 μm for (**A**).

To identify the optimal culture conditions, we compared three different culture conditions to identify the optimal culture environment. E8-CM group, E8 group, and TCP-CM group. Light microscopy revealed that the TCP-CM group formed spheroids of varying sizes, which lacked adhesion and exhibited instability, frequently wobbling and floating. In contrast, the E8 and E8-CM groups consistently produced small, homogeneous, and adherent 3D-CSC spheroids at the bottom of the PDMS microwells (Figure 3A). The qPCR results revealed that, compared to the pre-spheroid (TCP group), the expression of stem cell marker genes NANOG, PAX6, SOX2, OCT4, and KLF4 were upregulated by 3.72-fold, 2.89-fold, 4.61-fold, 3.08-fold, and 80.63-fold, respectively, in the E8-CM group (*p* < 0.05). When compared to the TCP-CM group, the E8-CM group showed up-regulation of NANOG, PAX6, SOX2, and KLF4 by 2.67-fold, 1.40-fold, 5.92-fold, and 1.50-fold, respectively (*p* < 0.05). There was no significant difference in the expression of OCT4 between the two groups (*p* = 0.24). Compared to the E8 group, the E8-CM group demonstrated upregulation of NANOG, SOX2, and KLF4 by 1.72-fold, 1.98-fold, and 5.81-fold, respectively (*p* < 0.05). In contrast, compared to pre-sphere formation, the expression of quiet cornea keratocyte genes (ALDH3A, CD34, and Keratocan) were downregulated by 0.06-fold, 0.01-fold, and 0.12-fold, respectively. At the same time, neural crest stem cell markers (Nestin and NGFR) were upregulated by 4.16-fold and 6.76-fold, respectively (*p* < 0.05) (Figure 3B–E).

Collectively, these findings indicate that the PDMS microwell platform effectively facilitates the formation of adherent 3D-CSCs. Additionally, spheroid culture on PDMS microwells enhances CSC stemness, with E8-CM further promoting their stem-like properties.

### 3.3. Stem Cell Characteristics in 3D-CSCs

**Figure 4:**
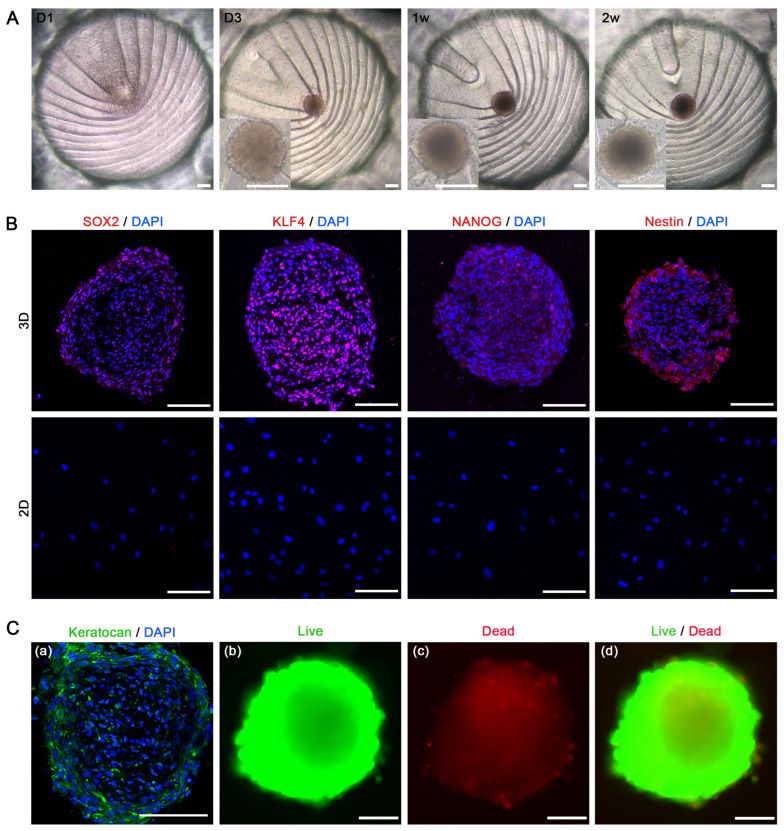
The stem cell characteristics of 3D-CSCs cultured in the PDMS microwell platform. (**A**) Bright-field images illustrating the morphological development of 3D-CSCs over time. hCSCs formed aggregates at the bottom of the PDMS microwells on day 1, developed a 3D structure by days 2–3, and maintained spheroid morphology throughout the 2-week culture period. (**B**) IF staining revealed that stem cell-related genes (KLF4, NANOG, SOX2, and Nestin, marked red) were positively stained in 3D-CSCs but negatively in 2D-CSCs. (**C**-**a**) IF staining revealed that the keratocyte marker (Keratocan, marked green) was positively stained in 3D-CSCs. (**C**-**b**,**c**,**d**) Live/dead staining of 3D-CSCs, using Calcein AM, showing green for live cells and red for dead cells. Scale bars: 100 μm for (**A**–**C**). *n* = 3 for (**B**,**C**).

Light microscopy was used to observe the morphological changes in 3D-CSCs on PDMS with E8-CM over time. On day 1, hCSCs began to aggregate at the bottom of the PDMS microwells, with a few cells adhering to the side walls. By days 2–3, the cells spontaneously formed 3D spheroids with well-defined boundaries. Gradually, they increased in volume and stabilized in morphology, eventually reaching a mean diameter of 236.02 ± 19.89 µm, as measured using a light microscope after two weeks of culture (Figure 4A). Throughout the culture, the spheroids remained confined within their respective microcavities, slightly adhering to the microwell bottom while sharing the same culture medium (E8-CM) and microenvironment. To investigate the stemness characteristics of 3D-CSCs, we compared them with 2D-CSCs (before spheroid culture). Immunofluorescence (IF) staining revealed that 3D-CSCs expressed key stemness markers, including Klf4, Nanog, Sox2, and Nestin, whereas 2D-CSCs showed no detectable expression (Figure 4B). IF staining demonstrated positive expression of the keratocyte marker (Keratocan) in the 3D-CSCs. This finding confirms that the 3D-CSCs are indeed corneal stromal cell spheroids. Live/dead cell staining demonstrated that the majority of 3D-CSCs were viable, exhibiting green fluorescence with Calcein AM, whereas only a small proportion of dead cells, marked by red fluorescence, were dispersed at the center and periphery of the spheroids (Figure 4C). These results suggest that the spheroid culture system using the PDMS microwells and E8-CM is a promising and safe approach for promoting cellular pluripotency and enhancing stemness.

### 3.4. Bioinformatics Analysis of 3D-CSCs

**Figure 5:**
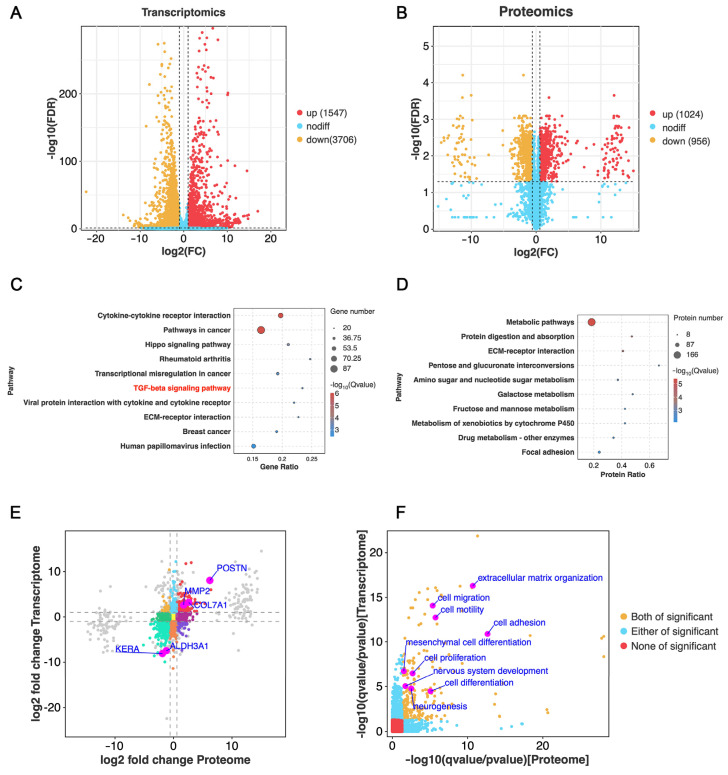
Transcriptomics and proteomics analysis of 3D-CSCs. (**A**,**B**) Volcano plots showing DEGs and DEPs, respectively. (**C**,**D**) The top 10 significantly altered KEGG pathways based on upregulated DEGs and proteins in the 3D-CSC group, the TGF-β signaling pathway (indicated in red) is associated with cell proliferation and differentiation. (**E**) Scatter plot of RNA-seq expression versus proteome expression in the nine quadrants. (**F**) GO term analysis for co-upregulated biological processes significantly enriched in both transcriptomic and proteomic data.

**Figure A1:**
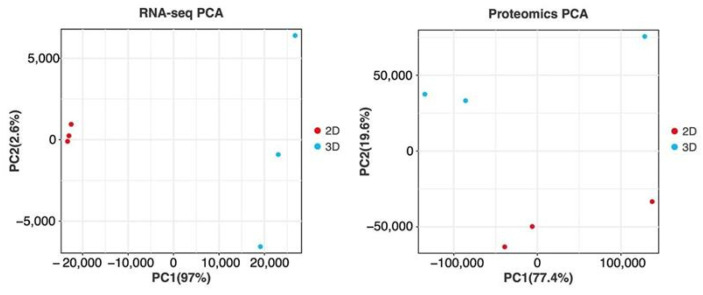
PCA of transcriptome and proteomics.

To further elucidate the molecular mechanisms underlying the advantages of spheroid culture, we conducted bulk RNA sequencing and proteomic analyses comparing 2D-CSCs (2D group) and 3D-CSCs cultured in E8-CM (3D group). Differentially expressed genes (DEGs) were identified based on a screening threshold of |Fold Change| > 2 and false discovery rate (FDR) < 0.05, while differentially expressed proteins (DEPs) were selected using |Fold Change| > 1.5 and FDR < 0.05. Volcano plots illustrated the global transcriptomic and proteomic changes. The Principal Component Analysis (PCA) diagram is shown in Appendix B (Figure A1). RNA-seq analysis identified 1547 upregulated and 3706 downregulated DEGs, whereas proteomic analysis revealed 1024 upregulated and 956 downregulated proteins (Figure 5A,B, more data in Supplementary Tables S1 and S2). Pathway analysis based on the KEGG annotation indicated significant enrichment in pathways associated with cell proliferation and differentiation, including cytokine-cytokine receptor interaction, TGF-beta signaling, ECM-receptor interaction, PI3K-Akt signaling, focal adhesion, and Wnt signaling (Figure 5C,D). A transcriptome-proteome integrative analysis revealed that genes and proteins in the upper right quadrant of a nine-quadrant diagram were significantly upregulated, suggesting their pivotal role in relevant biological processes, while those in the lower left quadrant were significantly downregulated, indicating suppressed expression (Figure 5E). GO term enrichment analysis further confirmed significant alterations in biological processes related to extracellular matrix organization, cell adhesion, mesenchymal stem cell (MSC) differentiation, stem cell development, cell proliferation, cell differentiation, nervous system development, neurogenesis, regeneration, cell migration and motility (Figure 5F). These findings highlight substantial transcriptomic and proteomic differences between 2D-CSCs and 3D-CSCs, with the latter exhibiting enhanced stemness-related processes.

### 3.5. Enhanced Cell Differentiation in 3D-CSCs

**Figure 6:**
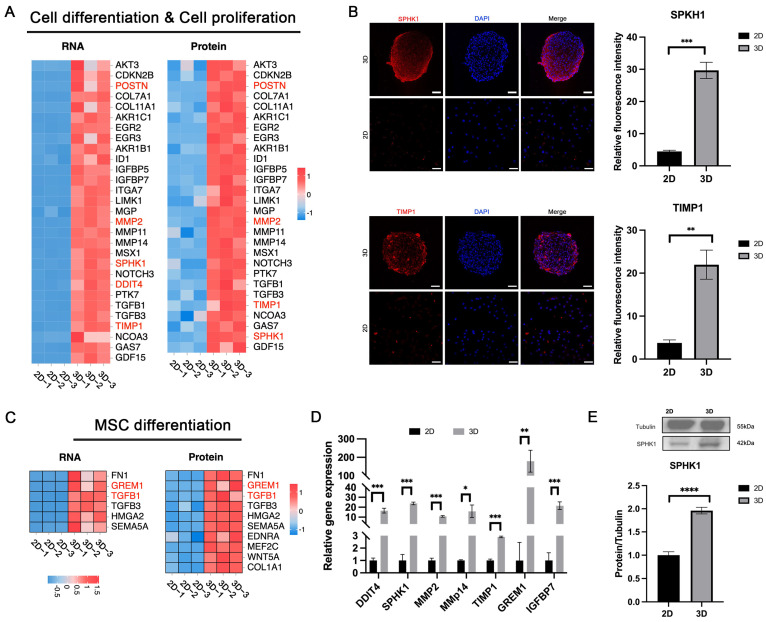
Cell differentiation and proliferation in 3D-CSCs. (**A**,**C**) Heatmaps showing cell differentiation, proliferation, and mesenchymal stem cell (MSC) differentiation markers in RNA-seq and proteomic data for 3D-CSCs. (**B**,**D**,**E**) Validation of cell differentiation and proliferation markers via qPCR, IF, and western blot (WB). The proliferation- and differentiation-related markers SPHK1 and TIMP1 show positive expression (marked in red) in the 3D group. Scale bars: 50 μm. Error bars represent standard deviations from at least three samples. (* *p* < 0.05; ** *p* < 0.01; *** *p* < 0.001; **** *p* < 0.0001; *n* = 3 for (**B**,**D**,**E**)). Original Western blot image of (**E**) can be found in Supplementary Materials File S1.

Following two weeks of spheroid culture, 3D-CSCs demonstrated significantly higher expression of genes associated with cell proliferation and differentiation, including MSC differentiation and regeneration, compared to the 2D group (Figure 6A–C). To validate these findings, we conducted qPCR, IF, and western blot (WB) analyses. qPCR confirmed significant upregulation of proliferation- and differentiation-related genes, including GREM1, TIMP1, SPHK1, MMP2, MMP14, DDIT4, and IGFBP7 in the 3D group (Figure 6D). IF staining showed stronger positive expression of TIMP1 and SPHK1 in 3D-CSCs, with significant differences in relative fluorescence intensity between the two groups (*p* < 0.05) (Figure 6B). Additionally, WB analysis confirmed that SPHK1 expression was significantly elevated in 3D-CSCs compared to 2D-CSCs (*p* < 0.05) (Figure 6E). These findings suggest that 3D-CSCs exhibited elevated cell proliferation and differentiation capacity.

### 3.6. Enhanced Nervous System Development in 3D-CSCs

**Figure 7:**
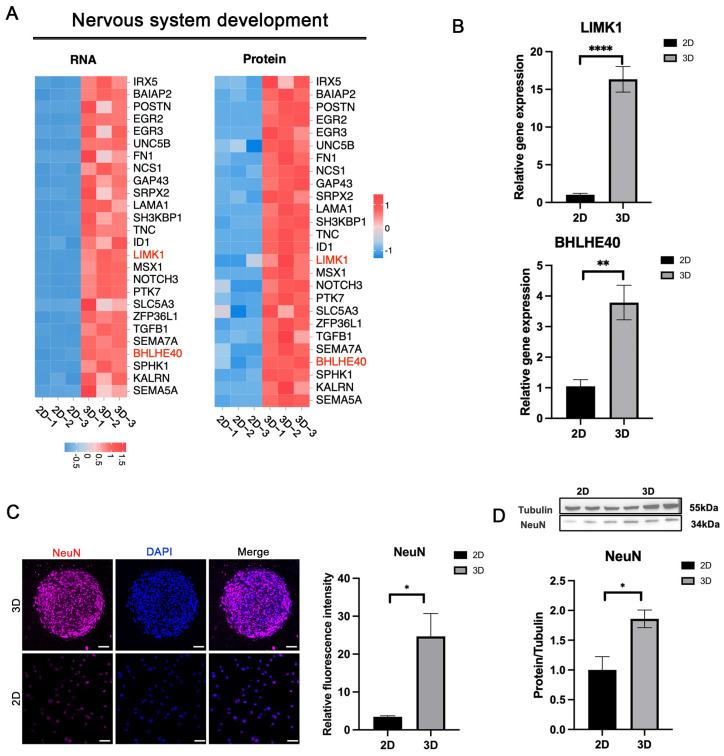
Nervous system development in 3D-CSCs. (**A**) Heatmaps depicting nervous system development-related factors in 3D-CSCs from RNA-seq and proteomic data. (**B**–**D**) Validation of nervous system development-related markers using qPCR, IF, and WB. The neuronal-specific marker NeuN (marked in red) shows positive expression in the 3D group. Scale bars: 50 μm. Error bars represent standard deviations from at least three samples. (* *p* < 0.05; ** *p* < 0.01; **** *p* < 0.001, *n* = 3 for (**B**–**D**)). Original Western blot image of (**D**) can be found in Supplementary Materials File S1.

RNA-Seq and proteomic analyses indicated that 3D-CSCs displayed higher expression of genes involved in nervous system development and neurogenesis (Figure 7A). qPCR validation confirmed significantly higher expression of neurogenesis-related markers BHLHE40 and LIMK1 in the 3D group (*p* < 0.05) (Figure 7B). Immunofluorescence staining demonstrated the neural crest cell marker NeuN-positive cells in 3D-CSCs, whereas the 2D group exhibited no detectable expression; the difference in relative fluorescence intensity between the two groups was statistically significant (*p* < 0.05) (Figure 7C). Additionally, WB analysis confirmed significantly higher NeuN protein expression in 3D-CSCs (*p* < 0.05) (Figure 7D). These results suggest that 3D-CSCs possessed greater pluripotency and neurogenic potential than the 2D group, indicating potential applications in neural tissue regeneration.

### 3.7. Enhanced Cell Migration and Adhesion in 3D-CSCs

**Figure 8:**
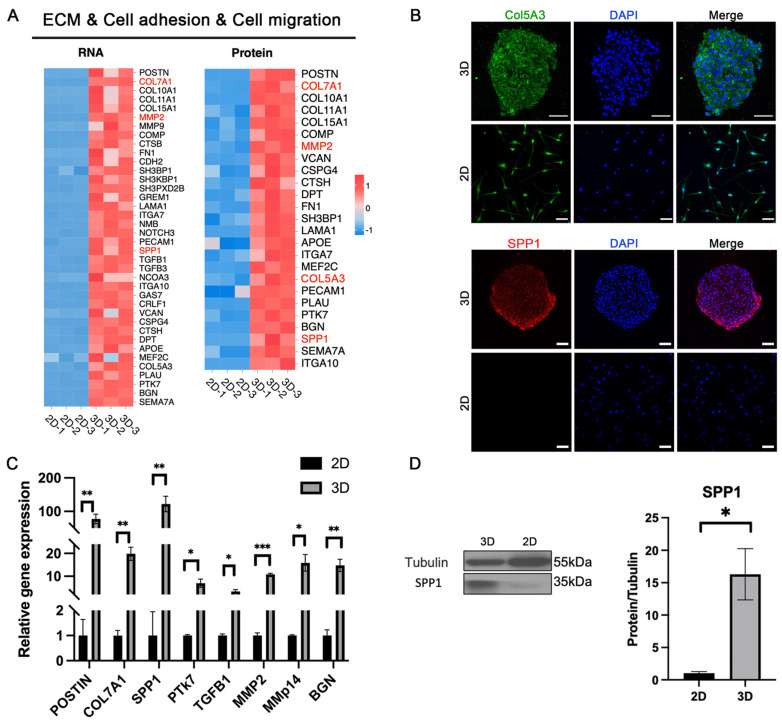
ECM organization, cell adhesion, and migration in 3D-CSCs. (**A**) Heatmaps illustrating ECM organization, cell adhesion, and migration-related factors in 3D-CSCs from RNA-seq and proteomic data. (**B**–**D**) Validation of ECM organization, cell adhesion, and migration markers via qPCR, IF, and WB. The ECM component COL5A3 (marked in green) and the adhesion-related marker SPP1 (marked in red) show positive expression in the 3D group. Scale bars: 50 μm. Error bars represent standard deviations from at least three samples. (* *p* < 0.05; ** *p* < 0.01; *** *p* < 0.001, *n* = 3 for (**B**–**D**)). Original Western blot image of (**D**) can be found in Supplementary Materials File S1.

We observed that biological processes such as extracellular matrix organization, cell migration, adhesion, communication, signal transduction, and positive regulation of cell migration were significantly enriched in the 3D group (Figure 8A). qPCR analysis confirmed that migration- and adhesion-related genes, including POSTIN, MMP2, MMP14, PTK7, BGN, SPP1, TGFB1, and COL7A1, were significantly upregulated in 3D-CSCs (Figure 8C). IF staining further demonstrated strong positive expression of the cell adhesion marker SPP1 in 3D-CSCs; the key ECM component, COL5A3, was positively expressed in 3D-CSCs, consistent with the proteomics results (Figure 8B). WB analysis corroborated these findings, showing significantly elevated expression of SPP1 in 3D-CSCs (*p* < 0.05) (Figure 8D). These results indicate that 3D-CSCs exhibited enhanced migratory and adhesive properties, which may facilitate their integration and functional role in tissue repair and regeneration.

## 4. Discussion

Clinical measures for the treatment of corneal opacity and scarring were not ideal. Cell therapy was an effective treatment for cornea diseases. However, the main challenge is finding seed cells with good viability and regenerative ability for cell therapy. At the same time, finding the best source of cells and in vitro culture has been the primary concern of researchers. In this study, hCSCs were collected from SMILE-derived lenticules, ensuring that the cells were healthy and fresh. Then, these cells were cultured in vitro in a 3D PDMS microwell with the E8-CM to promote cell stemness. There were three main findings in this study. First, we illustrated that the culture method of RIFA + hCSE medium helped to harvest high-quality, healthy human corneal stromal cells with favorable physiological phenotypes in vitro. Second, we obtained 3D-CSCs with a stem cell phenotype. Finally, the 3D-CSCs demonstrated significantly upregulated biological processes regarding cellular proliferation and differentiation, adhesion, migration, nervous system development, and MSC differentiation.

Our study provides a novel pathway to obtain high-quality and healthy CSCs in vitro. Our prior research demonstrated that quiescent keratocytes are more amenable to stemness than corneal fibroblasts [31]. Consequently, we focused on the quiescent subset of hCSCs in this study. Firstly, our study sourced hCSCs from a young and healthy human population. Traditional methods, which usually isolated CSCs from donated human corneal tissue or stem cells [24,45,46], were limited and challenging to scale. In contrast, we utilized discarded corneal lenticules from SMILE as new sources of hCSCs. These lenticules are uncontaminated by other cell types and provide a rich source of cells due to the increasing number of patients undergoing SMILE surgery, ensuring a consistent research supply. Secondly, we optimized the culture conditions. The physiological state of quiescent keratocytes was crucial for maintaining corneal transparency and function [6,47,48,49]. In this study, we innovatively supplemented human corneal extract (hCSE) to propagate CSCs. Compared with other animal ECM components, as natural and niche ECM components for CSCs, hCSE could provide a culture environment closest to human CSC survival in vivo. Results of IF and qPCR analysis showed that hCSCs cultured in the RIFA + hCSE medium expressed high levels of quiescent keratocyte stromal cell markers, such as ALDH3A1, CD34, KERA, and LUM, while exhibiting weak expression of fibroblast markers, such as Fibronectin and α-SMA. This indicated that our method provided an innovative solution for obtaining high-quality hCSCs.

In this study, we performed 3D spherical cultures of CSCs using a PDMS microwell platform. The PDMS platform was fabricated using 3D printing technology, creating 62 V-bottom microwells within a 6-well plate, where small and uniform 3D-CSCs were generated at the bottom of each well. By confining individual 3D-CSC aggregates within separate microcavities, we prevented the fusion of multiple aggregates and ensured the long-term culture of these cells. On the one hand, 3D cultures more accurately replicate the in vivo microenvironment compared to traditional monolayer cultures, enhancing anti-inflammatory and immunomodulatory properties and angiogenic capacity [16,17]. In Yen’s study, 3D MSC culture showed increased secretion of ECM molecules, including collagen I, IV, V, and VI, and Fibronectin, laminin, and perlecan, which can regulate stem cell fate [18]. The 3D MSC culture improved therapeutic applications by enhancing “drug signaling” and increasing homing and survival rates post-transplantation, partly due to protective mechanisms against oxidative stress and higher expression of VEGF-A and HIF-1α [17]. In spherical culture, both multiline-age-differentiating stress-enduring (Muse) cells and rabbit corneal stromal cells showed higher expression of stem cell markers, such as OCT3/4, SOX2, KLF4, NANOG, Vimentin, and CD34 were significantly upregulated [50,51]. Similar results were obtained in this study and exhibited that, compared to the pre-spheroid, the expression of stem cells-related transcription factors markers (NANOG, PAX6, SOX2, OCT4, and KLF4) were remarkably upregulated in post-spheroid. On the other hand, PDMS, as a flexible material, provided the physical and mechanical support for hCSCs to develop 3D-CSCs and mimic the microenvironment of cells in vivo. For example, Tai et al. introduced a novel 3D cell culture system that combines electrospun nanofibers with microfabricated PDMS patterns [52]. This system enables the controlled formation of semispherical human iPS colonies. They found that such a system could modulate shaping that effectively modulated the spatial distribution of mechano-sensitive mediators of iPS cells, affecting their subsequent differentiation behaviors [52]. Zhang et al. investigated the modulation of matrix stiffness on the phenotype and cell behavior of human induced pluripotent stem cell-derived corneal stromal cells (h-iCSCs). They found that softer PDMS (4 kPa) was more conducive to maintaining the phenotype of h-iCSCs [53]. Our outcomes were consistent with those reported studies. Hence, we applied PDMS as the material to construct the microwell platform. Additionally, we compared the 3D-CSCs generated on TCP with those generated on PDMS using the same E8-CM medium and found that the PDMS platform yielded superior results. Specifically, the 3D-CSCs generated on PDMS exhibited a faster spheroid-forming time, achieving uniform-sized spheroids within 2–3 days while adhering to the bottom of the microwell, with minimal scattered cells nearby. In contrast, the 3D-CSCs generated on TCP took approximately 5–7 days to aggregate into cell clusters and displayed significant morphological variation in size, with numerous scattered cells in the periphery. These TCP-derived spheroids were non-adhesive, making them prone to wobbling and drifting. Meanwhile, regarding stemness characteristics, the 3D-CSCs on PDMS had significantly higher gene expression levels of endogenous transcription factor genes OCT4, SOX2, KLF4, CMYC, and NANOG than those on TCP. The above results may partly explain why 3D-CSCs generated on PDMS enhanced stem cell properties more easily than those on TCP, highlighting the potential of 3D culture systems in promoting cell stemness.

To further enhance the stemness, we optimized the culture medium by supplementing it with an iPSC-derived conditioned medium (E8-CM) to further promote the expression of stemness phenotypes. Our results indicated that the expression levels of several stem cell genes in 3D-CSCs cultured with E8-CM were significantly higher than those cultured with E8 medium alone, suggesting that E8-CM was more beneficial. We believe stem cells and their paracrine factors, such as exosomes, have become valuable resources in regenerative medicine. Previously, Liang et al. reported that applying a conditioned medium of MSCs promoted the proliferation of human umbilical vein endothelial cells (HUVECs), enhanced tube formation, and stimulated energy metabolism through the ERK signaling pathway [54]. Myeongsik’s study mentioned that Exosomes from hiPSC alleviate aging in skin fibroblasts [55]. Studies have shown that stem cells and their paracrine factors can ameliorate the aging of skin fibroblasts [55] and significantly promote tissue repair and anti-aging effects in renal diseases [56]. Therefore, we believe that E8-CM exerts its effects primarily through the paracrine secretion of growth factors, cytokines, chemokines, and exosomes from iPS cells, which promote the proliferation, self-renewal, and differentiation of 3D-CSCs.

Here, the transcriptomic and proteomic investigations unveiled that multiple biological processes and signaling pathways associated with stem cells were upregulated in the 3D-CSCs compared to the 2D-CSCs. Notably, elevated levels of cell differentiation, cell proliferation, mesenchymal cell (MSC) differentiation, and regulation of tissue remodeling were noted in the 3D groups. The ability of Self-renewal, proliferation, and differentiation are the unique properties of stem cells. Our study revealed that both transcriptomics and proteomics analyses significantly enriched core signaling pathways associated with stem cell self-renewal. To be specific, the TGF-β and PI3K-Akt signaling pathways were significantly enriched in the transcriptomic and proteomic datasets, and the Wnt signaling pathway was prominently enriched in the transcriptomic analysis (more data in Supplementary Tables S3 and S4). We speculate that the enhanced stemness of 3D-CSCs is likely attributed to the activation of these signaling pathways. These signaling pathways could regulate gene expressions and maintain the undifferentiated state of cells [57,58,59]. As reported, Wnt signaling has emerged as an important factor in stem cell biology and is known to affect the self-renewal of stem cells in various tissues [57,60]. TGF-β signaling affects various processes, including cell proliferation, differentiation, and development [61,62]. In the future, we aim to conduct in-depth investigations into the underlying mechanisms.

At the same time, we observed that biological processes such as mesenchymal cell (MSC) differentiation and Nervous system development were significantly enriched in transcription and proteomics. These indications suggested that increasing cell differentiation and pluripotency occurred in the 3D-CSCs group. Through qPCR, IF, and WB experiments, it was confirmed that the cell proliferation, differentiation, and neurogenesis-related genes (GREM1, TIMP1, SPHK1, MMP2, POSTIN, BHLHE40, and LIMK1) were significantly upregulated. It has been demonstrated that GREM1 expression distinguishes unique connective tissue stem cells in both the bone and the intestine [63]; TIMP1 has various biological roles in regulating cell growth, apoptosis, differentiation, angiogenesis, and oncogenesis. It was a crucial survival factor that enhanced the survival of transplanted stem cell spheroids [64]. We also found that extracellular matrix organization, cell adhesion, and cell migration were significantly enhanced in the 3D group, which aligns with previous studies. In the future, we will further explore the specific roles and mechanisms of those related pathways or genes.

Cellular plasticity can be enhanced in adult cells to a pluripotent state by endogenous or exogenous factors [65,66,67,68,69,70]. Greene et al. reported that adult CSCs could be reprogrammed to a neuron-like cell using exogenous growth factors along with the increase in gene expression regulation of the structure and function of neurogenesis and mature neurons [9]. In contrast, non-genetic and exogenous factors, such as small chemical molecules, conditioned media, or physical methods, are simpler, easier, flexible, ideal, and safer ways to manipulate cell fate [71]. Our study improved the stemness of adult hCSCs using a non-genetic program. There are few reports on the non-genetic methods in promoting the stemness of human CSCs. Our research has added to the story, and further improvement could be made on this basis.

Recent studies have made significant efforts to develop 3D corneal organoids from human iPSCs [72,73,74]. The organoids harbor cell clusters that resemble cells of the corneal epithelium, stroma, and endothelium, with subpopulations that capture signatures of early developmental states. Unlike the adult cornea, where the largest cell population is stromal, the organoids contain large proportions of epithelial and endothelial-like cells. The pluripotent cell clusters committed to epithelial cell lineage at 1 month; early corneal epithelial, endothelial, and stromal cell markers at 2 months; keratocytes as the largest cell population at 3 months; and a large epithelial cell population at 4 months. In contrast, our study focuses on the stromal component, and our 3D spheroid system provides a more accessible and efficient model for studying and applying corneal stromal cells. Therefore, the 3D corneal organoid system is more conducive to studying corneal development and diseases. Both systems have unique advantages and contribute to the field of corneal cell therapy. A potential method for corneal organoid transplantation could involve organoid dissociation at different time points followed by Fluorescence-Activated Cell Sorting or Magnetic-Activated Cell Sorting (FACS/MACS) to enrich for better purification of the cells to be transplanted.

This study’s limitation was that although we demonstrated the potential of the 3D culture system in promoting the stemness of hCSCs, it did not fully assess these cells’ long-term safety and effectiveness for potential clinical applications. Future studies should focus on the therapeutic potential of hCSCs further and explore these cells’ long-term safety and efficacy in clinical settings.

## 5. Conclusions

In conclusion, our study introduces an innovative method for fabricating human corneal stromal spheroids using a novel three-dimensional (3D) cell culture system. This system is based on a PDMS microwell platform featuring numerous V-bottom microcavities. The RIFA + hCSE medium facilitates the retention of quiescent morphological and phenotypic characteristics in human corneal stromal cells (hCSCs) derived from SMILE lenticules. These hCSCs successfully formed small, homogeneous, and adherent 3D spheroids within the V-bottom microcavities of the PDMS microwell platform, achieving pluripotency and a robust stem cell phenotype with uniform size, stable structure, and increased expression of key pluripotency factors, including OCT4, NANOG, SOX2, KLF4, and PAX6.

Moreover, treatment with iPS supernatant E8-CM significantly enhanced the stemness of the 3D-CSCs. Both transcriptomic and proteomic analyses demonstrated that the 3D-CSCs exhibit superior biological processes related to cellular proliferation, differentiation, adhesion, migration, and neurogenesis. This innovative approach not only effectively repurposes discarded SMILE lenticules but also provides valuable insights for corneal tissue engineering and regenerative medicine. It highlights the potential of this method for treating corneal opacities and other corneal diseases.

## 6. Patents

The human corneal stromal Lenticules from SMILE surgery in this study were collected in the Aier Eye Hospital Group (Changsha, Hunan, China). Written informed consent was obtained from all patients.

## Data Availability

The data presented in this study are all contained within the main body of this article.

## Supplementary Materials

The following supporting information can be downloaded at: https://www.mdpi.com/article/10.3390/biom15030438/s1, Table S1: All DEGs of 2D and 3D group; Table S2: All DEPs of 2D and 3D group; Table S3: Enriched pathways in the transcriptomic; Table S4: Enriched pathways in the proteomics. File S1: Original Western blot image.

## Abbreviations

The following abbreviations are used in this manuscript:

SMILE	Small Incision Lenticule Extraction
CM	conditioned medium
ECM	extracellular matrix
iPS	induced pluripotent stem cells
hCSCs	human corneal stromal cells
hCSE	human corneal stromal extract
CSSCs	corneal stromal stem cells

## Appendix A

**Table A1:** Primers used in RT-PCR experiments.

Genes	Forward (5′-3′)	Reverse (5′-3′)
GAPDH	GGAAGGTGAAGGTCGGAGTC	GATCTCGCTCCTGGAAGATGG
LUM	GCTTCAATCAGATAGCCAGAC	CAGCCAGTTCGTTGTGAGA
KERA	AACCTGACCCTTCTTGACCT	ACTGCATTGTATTGGCTGGT
CD34	CTACAACACCTAGTACCCTTGGA	GGTGAACACTGTGCTGATTACA
ALDH3A1	TGTTCTCCAGCAACGACAAGG	AGGGCAGAGAGTGCAAGGT
THBS1	TATAGCGACCCCATGTACCG	AGTCTTCCTGCCCTGAGTTG
FN1	AAGACCATACCCGCCGAATG	GGCATTTGGATTGAGTCCCG
OCT4	AGAGGCAACCTGGAGAAT	ATAGTCGCTGCTTGATCG
SOX2	GCACAACTCGGAGATCAG	CAGCGTGTACTTATCCTTCT
KLF4	TGAACTGACCAGGCACTA	TCATGTGTAAGGCGAGGT
NANOG	TCTCCAACATCCTGAACCT	GCGTCACACCATTGCTAT
PAX6	ACATCTGGCTCCATGTTGGG	ATAACTCCGCCCATTCACCG
NESTIN	GCACCTCAAGATGTCCCTCAG	CTCCAGCTTGGGGTCCTGAAAG
NGFR	ACAAGACCTCATAGCCAGCAC	TGCAGCTGTTCCACCTCTTGA

**Table A2:** List of primary antibodies.

Antibodies	Species	Supplier	Catalog Number
ALDH3A1	rabbit	Abcam	Ab76976
Lumican	rabbit	Abcam	Ab168348
Fibronectin	rabbit	Abcam	Ab268020
α-SMA	rabbit	Bioss	bs-10196R
Nanog	rabbit	Abcam	Ab21624
Sox2	rabbit	Abcam	Ab97959
Klf4	rabbit	Abcam	Ab215036
Nestin	mouse	Santa Cruz	Sc-23927
NeuN	rabbit	Abcam	Ab177487
SPKH1	rabbit	Proteintech	10670-1-AP
SPP1	rabbit	Abcam	Ab214050
TIMP1	rabbit	Abcam	Ab211926
