# Supplementary figures and images for: Efficient Fabrication of Human Corneal Stromal Cell Spheroids and Promoting Cell Stemness Based on 3D-Printed Derived PDMS Microwell Platform
Source: Biomolecules. 2025 Mar 19;15(3):438. doi: 10.3390/biom15030438 (PMC11940411; doi:10.3390/biom15030438)

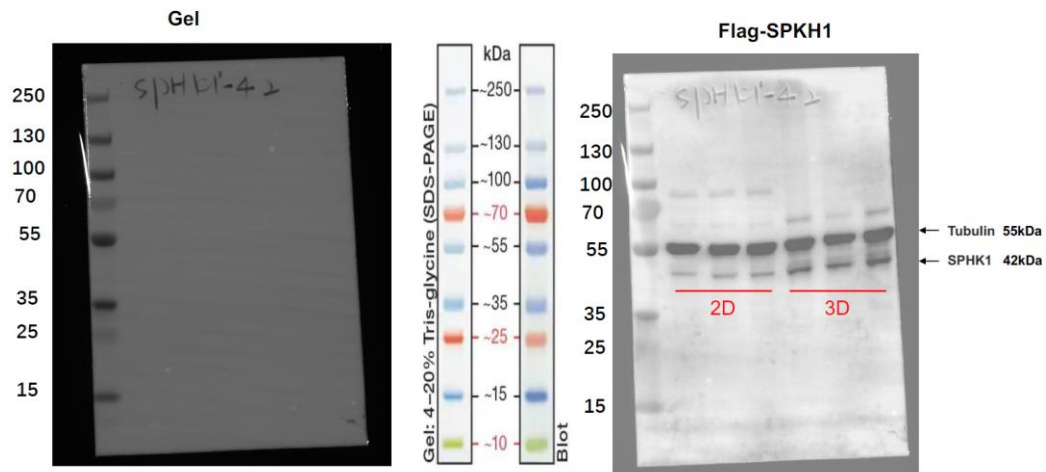

Figure 6E

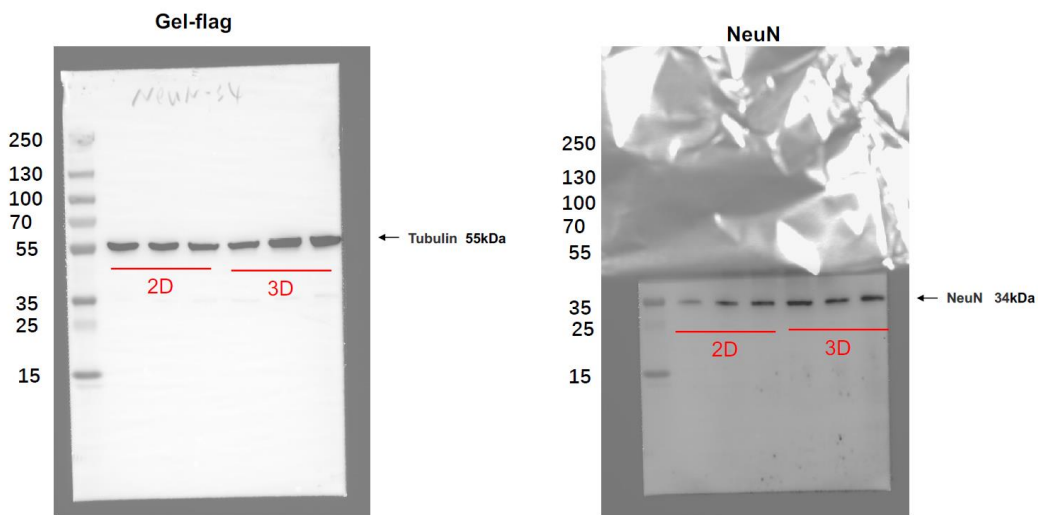

Figure 7D

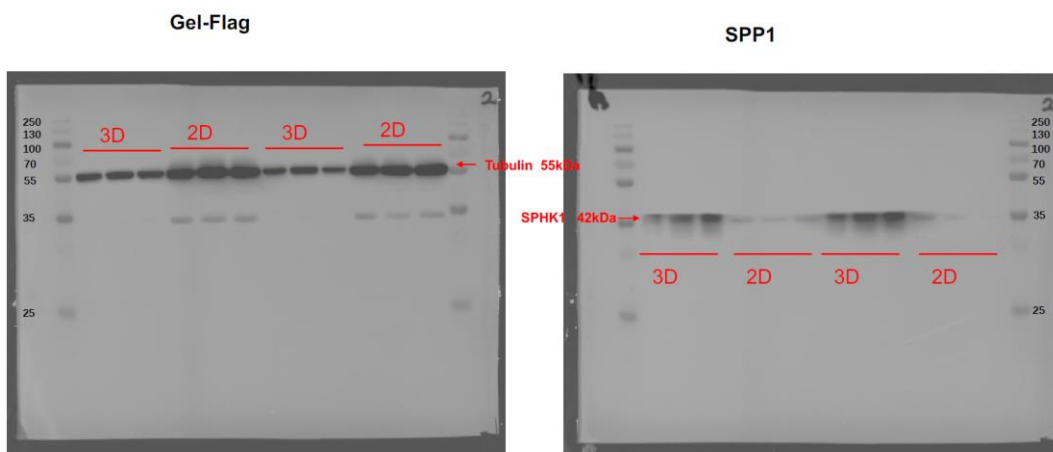

Figure 8D

Supplement: Supplementary file 1 [file biomolecules-15-00438-s001.zip › File S1.pdf]
